# Development of an Efficient FRET-Based Ratiometric Uranium Biosensor

**DOI:** 10.3390/bios13050561

**Published:** 2023-05-19

**Authors:** Sandrine Sauge-Merle, Morgane Recuerda, Maria Rosa Beccia, David Lemaire, Rym Cherif, Nicolas Bremond, Fabienne Merola, Yasmina Bousmah, Catherine Berthomieu

**Affiliations:** 1 Aix Marseille Université, CEA, CNRS, BIAM, UMR7265, IPM, 13108 Saint Paul-Lez-Durance, France; 2 Université Côte d’Azur, CNRS, Institut de Chimie de Nice, UMR 7272, 06108 Nice, France; 3 Université Paris-Saclay, CNRS, Institut de Chimie Physique, 91405 Orsay, France

**Keywords:** genetically encoded biosensor, uranium, FRET, sensing, sensitivity, selectivity, calmodulin, protein engineering, metal-binding

## Abstract

The dispersion of uranium in the environment can pose a problem for the health of humans and other living organisms. It is therefore important to monitor the bioavailable and hence toxic fraction of uranium in the environment, but no efficient measurement methods exist for this. Our study aims to fill this gap by developing a genetically encoded FRET-based ratiometric uranium biosensor. This biosensor was constructed by grafting two fluorescent proteins to both ends of calmodulin, a protein that binds four calcium ions. By modifying the metal-binding sites and the fluorescent proteins, several versions of the biosensor were generated and characterized *in vitro*. The best combination results in a biosensor that is affine and selective for uranium compared to metals such as calcium or other environmental compounds (sodium, magnesium, chlorine). It has a good dynamic range and should be robust to environmental conditions. In addition, its detection limit is below the uranium limit concentration in drinking water defined by the World Health Organization. This genetically encoded biosensor is a promising tool to develop a uranium whole-cell biosensor. This would make it possible to monitor the bioavailable fraction of uranium in the environment, even in calcium-rich waters.

## 1. Introduction

Uranium occurs naturally on earth, and its concentration can be locally increased in the environment by human activities associated with the nuclear industry, armament, or agriculture with the use of fertilizers. Uranium has a double chemical and radiological toxicity, and in cases of contamination, it can fix in certain organs and tissues, such as the kidney and bones, leading to pathologies such as kidney damage or bone cancers [1,2,3].

As part of environmental monitoring, it is important to measure the uranium content in soil and water in order to assess the risks to ecosystems and people and then propose protection countermeasures when necessary. The World Health Organization (WHO) has defined a concentration limit not to be exceeded in drinking water equal to 30 µg/L or 0.126 µM [4]. Current methods for measuring the uranium concentration in solution are analytical methods such as inductively coupled plasma mass spectrometry (ICP-MS), time-resolved laser fluorescence spectroscopy (TRLFS) and alpha emission. These methods are very sensitive, with detection limits of 1 to 2 ng/L (4.2 to 8.4 pM). However, they require laboratory measurements with expensive instruments, sample preparation and qualified technicians, and the results of these measurements are obtained after a few days. They do not allow the direct monitoring of uranium in the field. Another limitation of these methods is that they measure the total amount of uranium and not its toxic fraction. Indeed, this metal has a complex chemical speciation, meaning that uranium exists in different chemical forms in a given environment. Some of them are insoluble and are not available to living organisms and, hence, not toxic [5]. It would be interesting to measure only the fraction of uranium available to living organisms, called the bioavailable fraction, which represents the amount of metal that could be harmful to living organisms. 

In order to obtain uranium detection systems that are fast, inexpensive, easy to use and directly usable in the field, some biosensors have been developed. A biosensor is an analytical device that detects a specific analyte (uranium for instance) by combining a biological component (protein, antibody, DNA, bacterium) with a physicochemical detector [6,7]. There are a few examples of uranium biosensors in the literature. Some operate *in vitro*, such as monoclonal antibodies or catalytic DNA (DNAzymes). Monoclonal antibodies recognize uranium complexed to the 1,10-phenanthroline-2,9-dicarboxylic acid (DCP) with a detection limit of 900 pM to 10 pM [8,9,10]. Catalytic DNA was shown to cause DNA cleavage in the presence of uranium, with a detection limit of 45 pM [11]. These biosensors have a very good detection limit. However, their selectivity towards other metals could be improved, and in particular, their use in field conditions may be complex. Two interesting studies have been carried out to detect uranium *in situ* in groundwater. One consists of a repurposed personal glucose meter, which allows uranium detection by aptamers DNAzymes, with a detection limit of 9.1 nM [12]. The second study uses monoclonal antibodies attached to gold nanoparticles coupled to a lateral flow strip system and has a detection limit of 36.38 nM [13]. However, these biosensors cannot be used *in vivo* to assess the bioavailable toxic fraction of the metal for a given organism. The only way to achieve this is to use biosensors based on intact cells or organisms, called whole-cell biosensors [14,15,16,17]. They function as follows: the genetically encoded biosensor is expressed in an organism (bacterial cell, yeast, plant, etc.) and the fraction of the toxicant that enters this organism will trigger the biosensor signal. This makes it possible to quantify the bioavailable fraction of the toxicant, here, the bioavailable uranium content. The sole example of a uranium biosensor operating *in vivo* is a whole-cell biosensor resulting from the engineering of *Caulobacter crescentus* bacteria with a fluorescent reporter gene under the control of a uranium-inducible promoter. This biosensor shows a selectivity towards the four other compounds tested (lead, cadmium, chromium and nitrate) and its detection limit is 0.5 to 1 µM, a value above the uranyl concentration limit recommended by the WHO (0.126 µM) [18]. Recently, this biosensor has been modified and improved by integrating two independent two-component systems activated in the presence of uranium. The selectivity has been greatly improved with no cross reactivity with the most common environmental metals. However, the detection limit remained of the order of the micromolar, which may be due to the limited entry of uranium into the cell [19].

In nature, due to its complex chemical speciation, uranium occurs in several forms depending on its environment, but the main ones are the oxidized and soluble form U(VI) and a reduced form U(IV), which is not very mobile. In biological media, the hexavalent oxidation state U(VI) is predominant, almost exclusively in the form of the uranyl ion UO_2_^2+^ [20]. 

The mode of coordination of the uranyl ion is close to that of calcium ion: the nature of the ligands is similar with a preference for hard bases in Pearson’s classification [21], such as the oxygenated ligands carbonyl, carboxylate, phenolate or phosphoryl groups. The coordination number is also comparable since it is seven for calcium ion and five to six for the uranyl ion. The most common calcium-binding sites in proteins are the EF-hand motifs, which are structured by two α-helixes flanking a loop of twelve amino acids that carries the calcium ligands. This relatively flexible loop provides an interesting basis for creating binding sites for uranyl ions instead of calcium ions, as has already been successfully achieved on the whole calmodulin (CaM) protein or on its N-terminal domain [22,23,24,25]. In particular, we previously obtained an engineered uranyl-binding site with a subnanomolar affinity for uranyl and a very large selectivity towards calcium [26,27]. This prompted us to test whether we could use such EF-hand motifs of the CaM to develop an efficient uranyl biosensor.

A fluorescent calcium biosensor derived from the CaM has been developed by Tsien’s team [28]. This small protein binds four calcium atoms in four EF-hand motifs, located in dimeric arrangements in two distinct domains (N-terminal and C-terminal). Calcium binding causes a conformational change in the protein, which becomes more compact and adopts a dumbbell shape [29]. This conformational change motivated the design of a fluorescent cameleon-type biosensor, in which two fluorescent proteins (FPs) flank the sensing domain calmodulin, a fluorescence donor at the N-terminal and a fluorescence acceptor at the C-terminal. The compact shape adopted by CaM upon metal-binding brings the two FPs closer together, resulting in a Förster Resonance Energy Transfer (FRET) between the FPs [30]. The FRET value is used to determine the amount of metal bound to the CaM. The name cameleon comes from the fact that the biosensor is able to change color very quickly when metal is added. Miyawaki *et al.* demonstrated that it was possible to quantify the calcium content, with a detection limit of 10 nM [28]. A major advantage of this fluorescent biosensor is its ratiometric character, i.e., the measured FRET value, and thus, the determination of the amount of calcium is independent of the biosensor concentration.

Our objective is to develop a uranyl biosensor capable of quantifying the bioavailable fraction of this metal directly in water samples for environmental monitoring. In this work, we designed and characterized *in vitro* a genetically encoded fluorescent uranyl biosensor. Indeed, only a genetically encoded biosensor could then be expressed in a target host in order to transform this *in vitro* biosensor into an *in vivo* biosensor at a later stage [31,32]. We constructed this biosensor using an engineered CaM as the sensing domain by introducing the selective and sensitive uranyl-binding sites previously developed in our team in place of the calcium-binding sites [26,27]. In these previous studies, by engineering CaM calcium-binding site 1, we obtained a uranyl-binding site with subnanomolar affinity for uranyl and a more than 10^6^ uranyl to calcium selectivity. We also unambiguously showed that there is a direct interaction between uranyl and the CaM binding sites combining EXAFS, FTIR, molecular dynamics and fluorimetry. Here, we tested various variants that we characterized in vitro in terms of sensitivity (or affinity) and selectivity toward uranyl. We also tested two acceptor FPs to improve the biosensor’s robustness. The best combination resulted in an affine and selective biosensor for uranyl, no longer binding calcium, robust to environmental conditions, showing a good dynamic range and a promising detection limit below the limit value recommended by the WHO. 

## 2. Materials and Methods

### 2.1. Chemicals and Stock Solutions 

Chemicals were prepared with analytical grade salts (99.5%, Sigma-Aldrich, Saint-Quentin-Fallavier, France) dissolved in deionized water. The pH values of the solutions were measured as previously described [25].

### 2.2. Construction of Expression Vectors for Biosensors

Cloning steps were made with standard methods using *E. coli* XL1Blue cells (F’*::Tn10 proA^+^B^+^ lacI^q^* Δ*(lacZ)M15/recA1 endA1 gyrA96* (Nal^R^) *thi hsdR17 (rK^–^ mK^+^) glnV44 relA1 lac*). 

The synthetic gene (CaM-L-M13) coding for the calmodulin from *A. thaliana* fused with a five-residue linker, and the CaM-binding peptide of myosin light-chain kinase (M13) was purchased from Eurofins Genomics (see protein sequence in Table S1). It was cloned into the overexpression vector pQE30 (Qiagen) between the *Sac* I and *Sal* I restriction sites to obtain the plasmid A1. 

In the second step, the cDNA coding for the enhanced Yellow Fluorescent Protein (eYFP) was PCR-amplified using the primers S-eYFP-*Sal* I (TATAgtcgacatggtgagcaagggcgaggag) and AS-eYFP-*Hind* III (gggcaagcttttacttgtacagctcgtccatgccg) and cloned into the plasmid A1 downstream of the CaM-L-M13 gene, between the *Sal* I and *Hind* III sites, to give the plasmid A2. 

In the third step, the cDNA encoding the enhanced Cyan Fluorescent Protein (eCFP), containing the Tobacco Etch Virus protease (TEV protease) recognition site upstream of the coding sequence of eCFP, was PCR-amplified using the following primers: S-TEV-eCFP-*BamH* I (gAGAggatccgagaacctgtacttccagtccatggtgagcaagggcgaggag) and AS-eCFP-*Sac* I (TAAAgagctcggcggcggtcacgaactccagca). It was then cloned into the plasmid A2 upstream of the CaM-L-M13 gene, between the *BamH* I and *Sac* I sites, to give the final plasmid A3. 

Both genes contained no stop codon, except for the eYFP gene. The plasmid A3 contained the gene coding for biosensor 1 corresponding to the wild-type CaM sequence (Table S1). It was used as a template for new constructs carrying mutations. Site-directed mutations were performed using the QuickChange site-directed mutagenesis kit (Stratagene) following the supplier’s instructions. Information concerning the starting plasmids, primer pairs, and resulting plasmids with desired mutations are summarized in Table S2. 

### 2.3. Expression and Purification of the Biosensors

Protein expression and purification were performed as previously described [27], except that the cultures were incubated at 18 °C overnight after IPTG induction, the final purification step was performed on a Sephadex 200 size-exclusion column and the Microcon filtration system (Amicon Millipore^®^, Merck, Darmstadt, Germany) used to concentrate the proteins had a cut-off point of 30 kDa.

### 2.4. Mass Spectrometry Analyses

Mass spectrometry analyses were performed on a MicroTOF-Q (Bruker, Billerica, MA, USA) instrument with an electrospray ionization source, as previously described [27].

### 2.5. Fluorescence Measurements

The buffer solution (HEPES 50 mM pH 7) was incubated for at least 6 h with Chelex^®^-100 (Merck, Darmstadt, Germany) in order to remove any trace of divalent metals, such as calcium. The pH of the buffer solution was readjusted after this treatment because the Chelex^®^ causes a rise in pH. For the same reason, the protein solutions were incubated for 3–4 h with a 100-fold excess of ethylenediaminetetraacetic acid (EDTA). This step was used to remove calcium likely to be present in the different CaM binding sites. Then, the protein solutions were dialyzed overnight against the calcium-free buffer solution to remove divalent metal–EDTA complexes as well as free EDTA. We used only plastic laboratory equipment to avoid any possible calcium contamination from glass.

Fluorescence experiments were performed using an Infinite 1000 (TECAN, Männedorf, Switzerland) or a BioTek Synergy H1 Hybrid Reader (Agilent, Santa Clara, CA, USA) in microplate at 25 °C. Each buffer was treated with Chelex^®^-100 to remove all traces of calcium or other divalent cations. 

For each measurement for calcium or uranyl titrations, 0.5 or 1 µM of protein was mixed in 200 µL of 50 mM HEPES pH 7 buffer or in Mont Roucous mineral water (140 µM Na^+^, 67.5 µM Ca^2+^, 23 µM SO_4_^2−^, 12.3 µM Mg^2+^, 29 µM NO^3−^, 100 µM HCO^3−^ pH 6). For calcium titration, calcium nitrate was added at concentrations ranging from 0 to 80 µM. For uranyl titration, 10 µM of iminodiacetate (IDA, Merck, Darmstadt, Germany) was added to the buffer to control uranyl speciation and prevent its precipitation as hydro-uranyl complexes, which are formed at pH higher than 4, as described by Pardoux *et al.* in 2012 [23]. Then, uranyl nitrate was added at concentrations ranging from 0 to 25 µM. For each measurement for the potassium chloride titration, with concentrations ranging from 0 to 593 mM, 1 µM of protein was mixed in 200 µL of 30 mM CAPS, 30 mM MES and 30 mM Bis-Tris propane buffer adjusted at pH 7.4 by addition of either H_2_SO_4_ or NaOH (avoiding any chloride). For each measurement for the pH titration, 1 µM of protein was mixed in 200 µL of 50 mM citrate or 30 mM MCBTP, and the pH was adjusted with H_2_SO_4_ or NaOH from 2.5 to 11. Excitation was performed at 440 nm, and the emission spectrum was recorded between 450 and 570 nm. The recorded fluorescence spectra were normalized at 513 nm [33].

### 2.6. Dissociation Constants Calculations

To evaluate the affinity constants of calcium and uranyl ions for the proteins, each metal–protein complex formation was described using the total reaction:

K_cond_

4M +P ⇄ M_4_P (1)

where M is the metal ion (calcium or uranyl), P is the four-site protein and M_4_P is the final complex. K_cond_ is the association constant of the equilibrium, defined as:

K_cond_ = [M_4_P]/([M]^4^·[P]) (2)

where [M_4_P], [M] and [P] are the complex concentration, the free (non-complexed) metal concentration and the free peptide concentration, respectively. 

The assumption was made in the fit model that the four binding sites have equal affinity to the metal ion. Moreover, for the fit procedure, only experimental points at C_M_ ≥ 4C_P_ were taken into account (with C_M_ and C_P_ being the total analytical concentrations of the metal and the peptide, respectively). This allows us to neglect the concentrations of the 1:1, 1:2 and 1:3 complexes compared to the 1:4 complex. Therefore, the fluorescence binding isotherms were fitted to a model described by the Equation (3):

ΔF/C_P_ = Δϕ K_cond_ [M]^4^ /(1 + K_cond_ [M]^4^) (3)

where ΔF is the change in fluorescence signal during titration, C_P_ is the total protein concentration, Δϕ is the amplitude of the binding isotherm, K_cond_ is the conditional association equilibrium constant of reaction defined above and [M] is the free metal concentration [34]. For the analysis of calcium binding, experimental data were analyzed by means of analytical non-linear least-square fitting procedures performed using the Jandel Table Curve^TM^ package. 

For the analysis of uranyl binding, uranyl–IDA complex formation was taken into account. IDA chelates uranyl, forming three different complexes for which the stability constants at 25 °C and I = 0.1 M are known (Jiang *et al.* [35]). Therefore, the stability constants of the three uranyl–IDA complexation equilibria, together with the dissociation constant of IDA, also reported in Jiang et al. [35], were included in the calculation. When considering all the above equilibria in solution, the analytical estimation of the free M concentration [M] and, consequently, of K_cond_ is not possible. Therefore, [M] and K_cond_ were calculated for each binding isotherm with a Newton–Raphson numerical method based on the best fit of the measured fluorescence signal with respect to synthetic fluorescence signals computed numerically, using an algorithm previously implemented in Beccia *et al.* [25]. In this algorithm, the best fit is obtained through an iterative process, based on a gradient descent of the distance between the experimental and synthetical fluorescence curves. First, we input the experimental values of C_M_, C_P_, C_IDA_, pH and ΔF/C_P_ measured along the titration. Then, we initialize the algorithm with a first guess of the K_cond_ constant. This allows us to compute the corresponding speciation curves for all species in solution and, from that, the associated synthetical fluorescence curve. Finally, we adjust the values of the reaction constant so that the synthetical fluorescence curve matches the experimental one, through a gradient descent, until we reach convergence. We ensure that the obtained fit solution is independent from the initialization. The best fit enables us to identify the conditional equilibrium constant, K_cond_, together with the parameter Δϕ. The obtained values of K_cond_ allow us to evaluate the complex dissociation constant of each binding site as Kd = 1/(K_cond_)^1/4^. The values of Kd for the binding of calcium and uranyl to the investigated peptides are reported in Table 1. Considering that the four binding sites have equivalent affinity for the metal cation certainly remains a hypothesis that does not necessarily correspond to the actual coordination process. Nonetheless, this approximation remains useful for assessing, as a first approximation, the relative affinity of the various mutants for both calcium and uranyl.

## 3. Results

### 3.1. Characterization of Uranyl-Binding Properties of the CaM Used as a Template for the Biosensor

**Figure 1:**
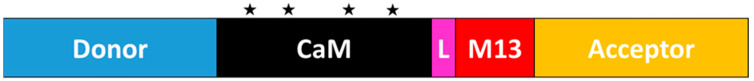
Schematic representation of the different biosensors. The donor is eCFP; CaM is the calmodulin of *Arabidopsis thaliana* with four metal-binding sites shown by ★ with or without mutations in one, two, three or four binding sites; L is a linker of five amino acids; M13 corresponds to the CaM-binding peptide of myosin light-chain kinase; the acceptor is eYFP or Citrine.

We chose to develop a genetically encoded uranyl fluorescent biosensor based on the work of Miyawaki *et al.* on the calcium biosensor [28]. To build biosensor 1, we used the whole CaM sequence of *Arabidopsis thaliana* coupled to a CaM-binding peptide of myosin light-chain kinase (M13), via a five amino acid linker (L). We inserted the enhanced Cyan Fluorescent Protein (eCFP) upstream of the CaM sequence and the enhanced Yellow Fluorescent Protein (eYFP) downstream of the M13 sequence (Figure 1, biosensor 1 in Table S1). The recombinant biosensor was produced in the *E. coli* cells. For purification, a His-tag, followed by the sequence of the TEV protease, was inserted at the N-terminus of the biosensor sequence. The TEV protease is a cysteine protease from Tobacco Etch Virus, which cuts the protein at the level of its recognition sequence after a glutamine residue. This results in an additional serine residue at the N-terminus in the purified proteins obtained by two steps of affinity chromatography and one step of size-exclusion chromatography. The mass spectrometry analysis showed that the purified biosensor was at the expected size, with, however, a small deviation from the theoretical mass due to the maturation, i.e., post-translational formation of the GFP’s chromophores [36]. 

**Figure 2:**
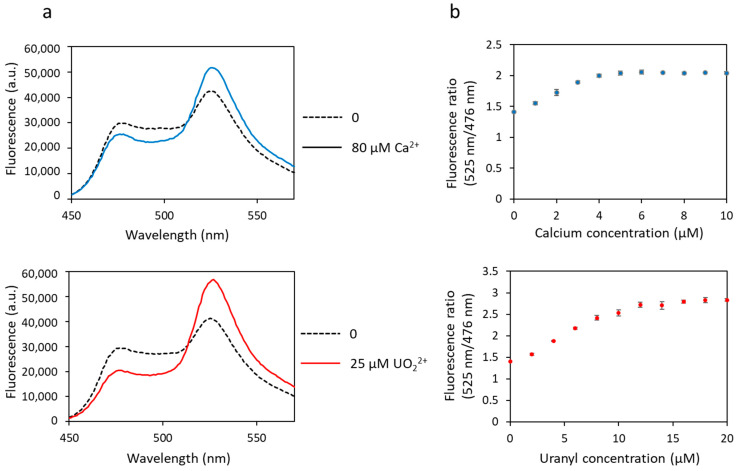
Fluorescence spectra of biosensor 1 with calcium or uranyl obtained with an excitation wavelength of 440 nm. (**a**) Fluorescence measured in arbitrary units (a.u.) with no metal (dashed lines) and 80 µM of calcium or 25 µM of uranyl (solid lines). (**b**) Fluorescence ratio obtained with fluorescence at 525 nm and at 476 nm as a function of the concentration of metal (calcium or uranyl). Measurements were performed in triplicate, and SD values are represented as error bars.

The affinity of biosensor 1 for calcium and uranyl was tested by measuring the fluorescence transfer between the two FPs. Iminodiacetate (IDA) was added to the protein solution to prevent precipitation of free uranyl, as previously described [23]. A basal level of FRET was observed in the absence of metal, as evidenced by the two fluorescence maxima observed at 476 nm and 525 nm (Figure 2a). Then, a decrease in fluorescence at 476 nm and a concomitant increase in fluorescence at 525 nm were observed following cation binding (calcium or uranyl) due to fluorescence transfer between both FPs (Figure 2a). This biosensor, inspired by the one previously developed for calcium, responds not only to calcium but also to uranyl, as shown in Figure 2. The maximum of FRET was obtained in the presence of ≥ 5 µM of calcium nitrate and ≥ 10 µM of uranyl nitrate (Figure 2b). 

The dynamic range of the biosensor response (∆R/R) (Equation (4)) was much larger for uranyl than for calcium, with 41% with calcium and 95% with uranyl (Table 1).

(4) $$\frac{\Delta R}{R}=\frac{R2-R1}{R1}*100=\left(\left(\frac{R2}{R1}\right)-1\right)*100$$

R2 is the maximum fluorescence ratio obtained by dividing the fluorescence at 525 nm by the fluorescence at 476 nm; R1 is the minimum fluorescence ratio obtained by dividing the fluorescence at 525 nm by the fluorescence at 476 nm when no metal was added.

The dynamic range depends on the nature of the FPs and the sensor part and on the distance between the two FPs and their respective orientation. It is crucial to have the highest possible dynamic range. It will increase the detection sensitivity of the FRET-based signal to small changes in metal amount. There are no dynamic range data in the literature for a genetically encoded uranium biosensor, but there are examples for calcium biosensors where the dynamic ranges vary between 10% and 1165% [37,38,39]. 

The dissociation constant (Kd) represents the affinity of the biosensor for a given metal: the lower the Kd, the higher the affinity. The Kd for biosensor 1 are given in Table 1. These values were obtained by measuring a global Kd for each protein, which was divided by four to give an average Kd value per site. The Kd is in the micromolar range for calcium (2.4 µM) and in the nanomolar range for uranyl (14.7 nM). Looking at Figure 2b, we could infer a micromolar Kd for uranyl, but this would be wrong. We have to take into account the competition with IDA, which decreases the amount of free uranyl, as mentioned previously.

These results show that biosensor 1 is functional with uranyl as well as with calcium, exhibits a correct dynamic range, larger for uranyl than for calcium, but it is not selective as it responds to both metals. This first version of the biosensor is, however, promising because it shows that binding of uranyl occurs and induces a FRET variation. Moreover, uranyl binding occurs for uranyl concentrations in the nanomolar range, i.e., with a detection sensitivity below the limit value defined by the WHO (0.126 µM or 126 nM).

### 3.2. Effect of CaM Site 2 Inactivation on FRET

**Figure 3:**
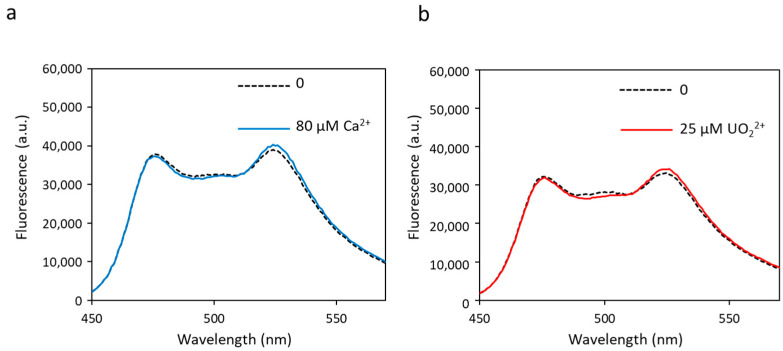
Fluorescence spectra of biosensor S2I with calcium (**a**) or uranyl (**b**) obtained with an excitation wavelength of 440 nm. Fluorescence measured in arbitrary units (a.u.) with no metal (dashed lines) and 80 µM of calcium or 25 µM of uranyl (solid lines).

In order to simplify the biosensor and reduce the number of metal-binding sites, we first tested mutations in site 2, aiming at preventing site 2 from binding any kind of metal. These mutations replace the two ligands of calcium (aspartate residues) at positions 1 and 3 of site 2 into alanine residues, leading to a new biosensor called biosensor S2I (sequence in Table S1). Fluorescence transfer experiments carried out on biosensor S2I showed no change in FRET, regardless of the amount of metal added (Figure 3). The dynamic range (∆R/R) was almost null (Table 1). This experiment demonstrates that metal-binding at site 2 is essential to generate a conformational change able to induce FRET. In the absence of functional site 2 and despite the presence of the three other metal-binding sites, the conformational change necessary to bring the two fluorescent proteins close together does not occur. This is in line with a previous model proposed by Tripathi and Portman in which site 2 has a higher flexibility and an earlier structural change than site 1 when binding metal, indicating that it is the metal-binding at site 2 that governs the open-to-closed conformational change in the CaM N-terminal domain [40]. In conclusion, site 2 must be functional in the biosensor, and its affinity for the cation of interest may be a limiting factor for the biosensor’s sensitivity.

### 3.3. Insertion of Engineered Uranyl-Binding Sites in the CaM Template of the Biosensor

To increase the selectivity of the biosensor towards uranyl, several mutations in the CaM sequence were tested. In a previous study focused on CaM site 1, we showed that it was possible to obtain a uranyl-selective and affine binding site [26,27] by deleting two amino acids of the calcium-binding loop. This deletion concerned the amino acids at positions 2 and 3 of the loop, including the calcium ligand Asp at position 3, and it was named ∆_2–3_ deletion. This resulted in a metal-binding site with a very high affinity for uranyl (Kd ≤ 0.2 nM) and a low affinity for calcium, with a Kd for calcium in the millimolar range (Kd = 8.7 mM) [27]. In this study, the ∆_2–3_ deletion was introduced in the different metal-binding loops of CaM, in one or more sites. This gave the following biosensors: biosensor ∆1 (∆_2–3_ deletion in site 1, the other sites as in the original sequence), biosensor ∆1∆3 (∆_2–3_ deletion in sites 1 and 3; sites 2 and 4 as in the original sequence), biosensor ∆1∆2∆3 (∆_2–3_ deletion in sites 1, 2 and 3; site 4 as in the original sequence) and biosensor ∆1∆2∆3∆4 (∆_2–3_ deletion in sites 1, 2, 3 and 4) (sequences in Table S1). 

#### 3.3.1. Effect of ∆_2–3_ Deletions on the Biosensor Response to Calcium

**Figure 4:**
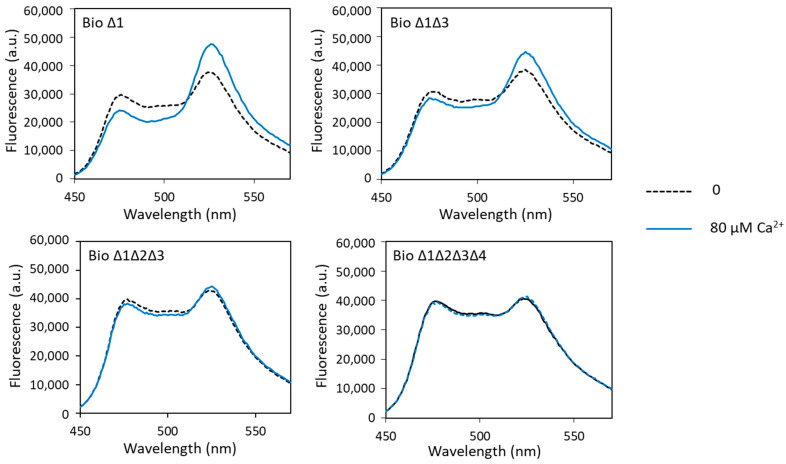
Fluorescence spectra of mutated biosensors with calcium obtained with an excitation wavelength of 440 nm. Fluorescence measured in arbitrary units (a.u.) with no metal (dashed lines) and 80 µM of calcium (solid lines).

Fluorescence analysis of these different biosensors in the presence of calcium showed calcium-induced FRET changes only with biosensor Δ1 and biosensor Δ1Δ3. For biosensor Δ1, the measured dynamic range was slightly higher than that of biosensor 1 (49% and 41%, respectively). The dynamic range decreased sharply with the increase in the number of sites carrying the ∆_2–3_ deletion (Figure 4, Table 1). It was only 28% when sites 1 and 3 had the ∆_2–3_ deletion, fell to 4% when sites 1, 2 and 3 had the ∆_2–3_ deletion and was almost null (2%) when all four metal-binding loops were mutated. These results show that sites 2, 3 and 4 play a major role in the calcium-dependent FRET variations in the calcium biosensor, in contrast to site 1, which does not seem to be important. Indeed, we have explained above that the presence of the metal-binding site 2 was decisive for the conformational change in the biosensor allowing FRET. Moreover, it is known in the literature that the C-terminal domain of CaM has a better affinity for calcium than the N-terminal domain, i.e., that sites 3 and 4 have a better affinity for calcium than sites 1 and 2 [41]. These results also show that the ∆_2–3_ deletion suppresses calcium binding not only at site 1, as previously demonstrated [26,27], but also at the other metal-binding sites of CaM.

#### 3.3.2. Effect of ∆_2–3_ Deletions on the Biosensor Response to Uranyl

**Figure 5:**
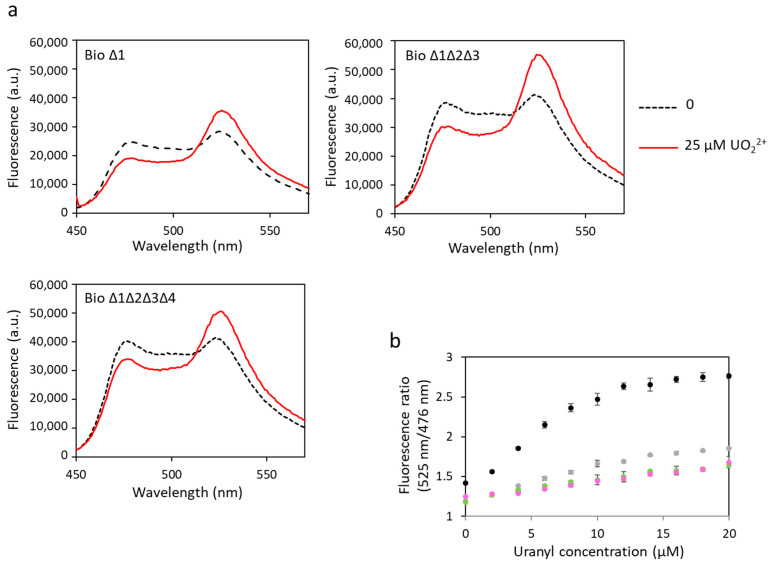
Fluorescence spectra of mutated biosensors with uranyl obtained with an excitation wavelength of 440 nm. (**a**) Fluorescence measured in arbitrary units (a.u.) with no metal (dashed lines) and 25 µM of uranyl (solid lines). (**b**) Fluorescence ratios obtained with fluorescence at 525 nm and at 476 nm as a function of the concentration of uranyl with biosensor 1 (●), biosensor ∆1 (●), biosensor ∆1∆2∆3 (●) or biosensor ∆1∆2∆3∆4 (●). Measurements were performed in triplicate, and SD values are represented as error bars.

The same biosensors have been tested to detect uranyl (Figure 5, Table 1). Changes in FRET were observed for all constructions, indicating uranyl binding to these biosensors. A decrease in dynamic range was observed in response to uranyl with the increase in the number of sites with ∆_2–3_ deletions. However, in contrast with the results obtained in response to calcium, significant FRET was still observed for biosensors with three or four mutated sites. The dynamic range of biosensor ∆1 was 56%, compared with 95% for the biosensor with no mutation (biosensor 1). We still observed a 41% dynamic range for biosensor ∆1∆2∆3 and 38% for biosensor ∆1∆2∆3∆4 in response to uranyl, *versus* 4% and 2%, respectively, in response to calcium. These results show that the ∆_2–3_ deletion inserted in each of the sites still allows the binding of uranyl. The Kd have not been calculated for these biosensors with Δ_2–3_ deletions. Indeed, we considered by simplification that the four sites have the same affinity for the metal in the calculation of the Kd of biosensor 1, and this is probably a false assumption in the case of biosensors including Δ_2–3_ deletion sites and wild-type sites. In addition, it was not possible to calculate a Kd for uranyl for biosensors with ∆_2–3_ deletions in one or several sites because the curves of fluorescence ratio *versus* metal concentration did not reach a plateau (Figure 5b). This also means that the affinity of these biosensors for uranyl is not as good as that of biosensor 1. However, their advantage is that they are selective for uranium with respect to calcium. 

The biosensor ∆1∆2∆3∆4 is very promising since it fixes uranyl with a correct dynamic range and no longer fixes calcium, i.e., it is affine for uranyl and selective for uranyl towards calcium. 

In addition, the ∆_2–3_ deletion was also performed in site 1 of a biosensor whose site 2 had been inactivated (Biosensor ∆1S2I, Table S1). Fluorescence analyses of this biosensor in the presence of calcium or uranyl gave the same results as for the biosensor S2I, i.e., no increase in the FRET signal corresponding to a dynamic range null or quasi null (Figure S2, Table 1). This result confirms the essential role of metal-binding at site 2 to induce FRET.

### 3.4. Robustness of the Biosensor

**Figure 6:**
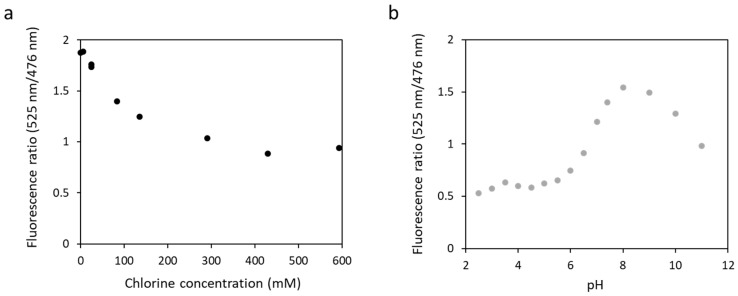
Fluorescence ratio obtained with fluorescence at 525 nm and at 476 nm for biosensor 1 as a function of chloride concentration at pH 7.4 (**a**) or as a function of pH ranging from 2.5 to 11 in the presence of 135 mM of chloride (**b**). The excitation wavelength was 440 nm.

Authors have reported that some yellow fluorescent proteins are sensitive to chloride ions [42] or pH [43], altering the fluorescence properties of the biosensors in the presence of large concentrations in salt. To test this, we measured the fluorescence of biosensor 1 in the presence of an increasing concentration of potassium chloride at pH 7.4 (Figure 6a). The fluorescence ratio decreased from 1.9 to 0.9 with an increase in chloride concentration from 0 to 593 mM. Biosensor 1 was then tested in a wide range of pH, from 2.5 to 11, in the presence of 135 mM potassium chloride (Figure 6b). The results obtained showed that the fluorescence ratio varied with pH. It increased from 0.5 to 1.5 at pH 2.5 to pH 8 and then decreased to 1 at pH 11. These results confirmed those obtained by other teams, showing a sensitivity of the eYFP to chloride ions or pH in our biosensor.

These data show that biosensors with an eYFP as a fluorescence acceptor cannot be used in the field with complex media or pH values other than physiological pH.

### 3.5. Improvement of the Biosensor Robustness and Selectivity

**Figure 7:**
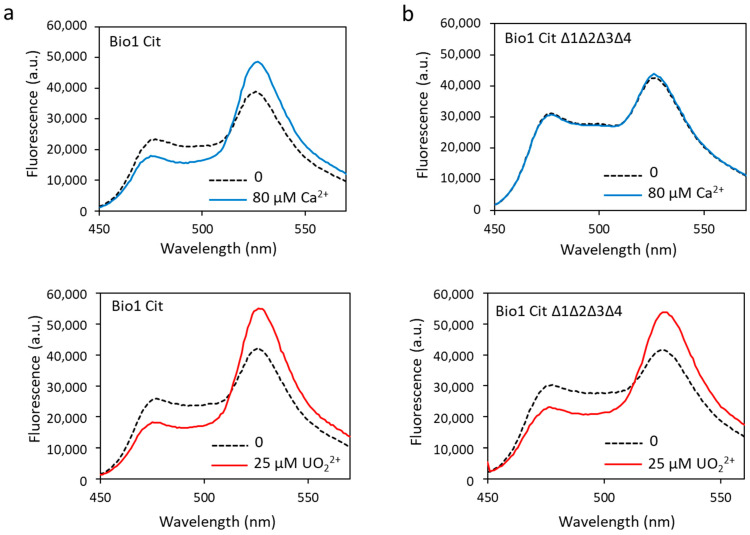
Biosensor Cit (**a**) and biosensor Cit ∆1∆2∆3∆4 (**b**) were analyzed in fluorescence with 80 µM of calcium or with 25 µM of uranyl with an excitation wavelength of 440 nm. Fluorescence measured in arbitrary units (a.u.) with no metal (dashed lines) and calcium or uranyl (solid lines).

To correct this problem, the eYFP was replaced by a variant of YFP, the Citrine protein, another acceptor FP that is not sensitive to pH and chloride ions [44]. The appropriate mutations were made in biosensor 1, i.e., the change of amino acids 69 and 70 of the eYFP (Val69Leu, Gln70Met) to produce the new construction, called biosensor Cit (Tables S1 and S2). The latter was then tested in the presence of calcium or uranyl. In addition to no longer being sensitive to pH and chloride ions (data not shown), the dynamic response range of biosensor Cit in response to calcium was increased (53% vs. 41%) and that in response to uranyl was almost equivalent to the one measured with biosensor 1 (82% vs. 95%) (Figure 7a, Table 1). The calculated Kd for biosensor Cit were almost equivalent to those for biosensor 1, with a Kd = 6.1 µM for calcium and a Kd = 14.2 nM for uranyl (Table 1).

To combine robustness and selectivity improvement, we engineered the biosensor Cit ∆1∆2∆3∆4 containing a ∆_2–3_ deletion in the four metal-binding sites of calmodulin and the Citrine protein. Fluorescence analysis of the biosensor Cit ∆1∆2∆3∆4 showed that in the presence of calcium, the dynamic response dropped drastically to 6% (Figure 7b, Table 1). As expected, this construction was no longer able to bind calcium ions. On the other hand, this new biosensor fixed the uranyl ions with a large response dynamic of 72% (Figure 7b, Table 1), significantly greater than that of biosensor Δ1Δ2Δ3Δ4 (38%). 

**Figure 8:**
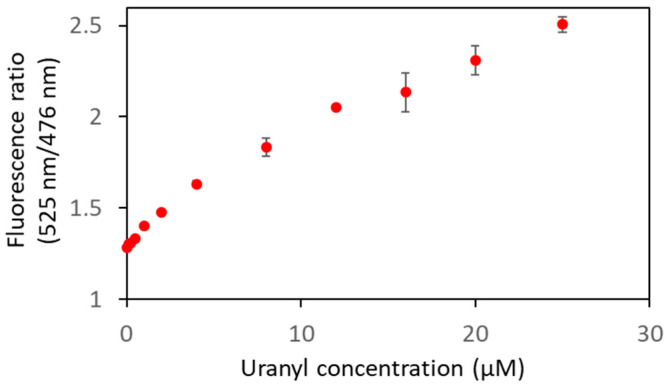
Biosensor Cit ∆1∆2∆3∆4 was analyzed in fluorescence in the presence of varying concentrations of uranyl with an excitation wavelength of 440 nm. The curve represents the fluorescence ratios (F_525nm_/F_476nm_) as a function of uranyl concentrations. Measurements were performed in triplicate, and SD values are represented as error bars.

To further characterize the biosensor Cit ∆1∆2∆3∆4, smaller concentrations of uranyl were used to determine its limit of detection (Figure 8, Table 2). A FRET variation was observed from 0.1 µM of uranyl ion, which is below the limit value defined by the WHO (0.126 µM).

Then, the biosensor Cit ∆1∆2∆3∆4 was tested in Mont Roucous mineral water, which has a richer mineralogical composition than a laboratory buffer (see composition in Materials and Methods). The dynamic range value is almost the same as that obtained in the HEPES buffer (70% *versus* 72%), indicating that the response of the biosensor is not disturbed by the presence of other metals such as calcium, sodium, magnesium, etc. (Table 1). In parallel, the biosensor Cit ∆1∆2∆3∆4 response was tested with sodium chloride, magnesium chloride or potassium chloride with concentrations up to 100 µM or 1 mM, and no basal FRET variation was observed (Table S3).

The biosensor Cit ∆1∆2∆3∆4 is thus very promising, since (i) it is able to bind uranyl ions with a good dynamic range, (ii) it is not sensitive to chloride ions or pH changes, (iii) it exhibits a high uranyl *versus* calcium selectivity, (iv) its limit of detection is below the limit value recommended by the WHO and (v) it works in a medium more complex than a laboratory buffer. 

## 4. Discussion

To identify the bioavailable fraction of uranyl in water, we need a genetically encoded uranyl biosensor with high affinity, selectivity and robustness that could be expressed in an organism. Towards this aim, in this work, we first studied the in vitro response to uranyl of a FRET-based ratiometric biosensor consisting of the whole CaM, flanked by two FPs, as described by Miyawaki et al. [28]. To our knowledge, this is the first genetically encoded uranium biosensor. With this construction, we showed that interaction with uranyl could induce FRET with a good dynamic range and that the affinity for uranyl was in the two-digit nanomolar range, *versus* a micromolar range for calcium. This was consistent with previous studies showing that the site 1 metal-binding loop of calmodulin has an affinity which is about 1000 times higher for uranyl than for calcium in vitro with a Kd of 32 nM for uranyl and 38 µM for calcium [23]. It further suggested that a higher affinity for uranyl than calcium exists also for other metal-binding loops. We recently showed that the affinity of site 2 for uranyl is in the hundreds of nM range (Kd ≈ 250 nM) [25]. The high affinity of sites 1 and 2 for uranyl probably explains the sensitivity of the biosensor obtained in the nanomolar range, indicating a detection threshold below the threshold value defined by the WHO. On this biosensor, we also showed that metal-binding at site 2, regardless of the nature of the attached metal, is primordial to induce FRET variation, a result not previously demonstrated in the literature. 

By deleting the two amino acids at positions 2 and 3 of the metal-binding loops in all metal-binding loops, we obtained a biosensor selective for uranyl towards calcium. The dynamic range was smaller in this engineered biosensor as compared to the initial one, but still significant and promising. In addition, comparison of the biosensors with three or four sites engineered with the ∆_2–3_ deletion indicated that uranyl binding at site 4 has only a modest effect on the FRET dynamic range (confers almost similar dynamic range values for these two mutants). Importantly, by substituting the receptor eYFP with a Citrine protein, we not only reinforced the robustness of the biosensor towards solutions with high chlorine concentrations or solutions at acidic or basic pHs, but we also increased the FRET dynamic range in response to uranyl. This biosensor, which combines the introduction of the ∆_2–3_ deletion in the four metal-binding sites and the substitution of eYFP in Citrine, proves to be affine for uranyl, and its detection limit is below the limit value of 0.126 µM recommended by the WHO. It is selective for uranyl towards calcium or other environmental metals, such as sodium or magnesium, since it works properly in the presence of these competitors or in Mont Roucous mineral water with a more complex mineral composition than laboratory buffers. It shows a good dynamic range, and because of its new acceptor FP, it should be robust to various environmental conditions. 

All these criteria are essential to develop an efficient biosensor which works in the field. Furthermore, the fact that the biosensors described here are genetically encoded encourages the development of whole-cell uranyl biosensors in different types of organisms (bacteria, plants, zebra fish) to test the bioavailable fraction of uranyl in water samples. 

## Figures and Tables

**Table 1:** ∆R/R calculated using Equation (4) for different biosensors in the presence of 80 µM of calcium or 25 µM of uranyl and average Kd calculated for one site (detailed in Materials and Methods, Figure S1) for some biosensors. These values resulted from the average of at least three independent experiments. ✕ Not tested; * not possible to determine. ^1^ Fluorescence was recorded in Mont Roucous mineral water instead of HEPES buffer 50 mM at pH 7.

Protein Name	∆R/R with Ca^2+^	∆R/R with UO_2_^2+^	Kd with Ca^2+^	Kd with UO_2_^2+^
Biosensor 1	41%	95%	2.4 µM	14.7 nM
Biosensor ∆1	49%	56%	*	*
Biosensor ∆1∆3	28%	✕	*	*
Biosensor ∆1∆2∆3	4%	41%	*	*
Biosensor ∆1∆2∆3∆4	2%	38%	*	*
Biosensor S2I	2%	4%	*	*
Biosensor ∆1S2I	0%	2%	*	*
Biosensor Cit	53%	82%	6.1 µM	14.2 nM
Biosensor Cit ∆1∆2∆3∆4	6%	72%	*	*
Biosensor Cit ∆1∆2∆3∆4 ^1^	✕	70%	*	*

**Table 2:** Fluorescence ratios (F_525nm_/F_476nm_) as a function of the concentration of uranyl. Measurements were performed with 1 µM of biosensor in 50 mM HEPES pH 7 with an excitation wavelength of 440 nm. The averages of the fluorescence ratios resulted from three independent experiments.

[Uranyl] in µM	Mean (F_525nm_/F_476nm_)	Standard Deviation
0	1.282	0.015
0.1	1.303	0.015
0.2	1.308	0.011
0.5	1.334	0.012
1	1.403	0.005
2	1.478	0.012
4	1.632	0.015
8	1.836	0.049
12	2.052	0.008
16	2.136	0.107
20	2.308	0.080
25	2.508	0.042

## Data Availability

All data needed to evaluate the conclusions in the paper are present in the paper and the Supplementary Materials. The raw data presented in this study are available on request from the corresponding author.

## Supplementary Materials

The following supporting information can be downloaded at https://www.mdpi.com/article/10.3390/bios13050561/s1, Table S1: List of protein sequences used in this study; Table S2: List of mutations, primer pairs, starting plasmids, resulting plasmids and resulting biosensors; Table S3: ∆R/R calculated for biosensor Cit ∆1∆2∆3∆4; Figure S1: Speciation diagram for a solution containing uranyl, IDA and biosensor 1 + fitting curves for biosensor 1 and biosensor Cit; Figure S2: Fluorescence spectra of biosensor ∆1S2I with calcium or uranyl.
