# Supplementary material for: Development of an Efficient FRET-Based Ratiometric Uranium Biosensor
Source: Biosensors (Basel). 2023 May 19;13(5):561. doi: 10.3390/bios13050561 (PMC10216315; doi:10.3390/bios13050561)
Supplement: Supplementary file 1 [file biosensors-13-00561-s001.zip › biosensors-2378453-supplementary.pdf]

Supplementary Materials

Development of an efficient FRET-based ratiometric uranium biosensor

Sandrine SAUGE-MERLE[1*], Morgane RECUERDA[1], Maria Rosa BECCIA[2], David LEMAIRE[1], Rym CHERIF[1], Nicolas BREMOND[1], Fabienne MEROLA[3], Yasmina BOUSMAH[3], Catherine BERTHOMIEU[1]

[1] Aix Marseille Université, CEA, CNRS, BIAM, UMR7265, IPM, 13108 Saint Paul-Lez-Durance, France

[2] Université Côte d'Azur, CNRS, Institut de Chimie de Nice, UMR 7272, 06108 Nice, France

[3] Université Paris-Saclay, CNRS, Institut de Chimie Physique, 91405 Orsay, France

[*]Corresponding author. E-mail: sandrine.sauge-merle@cea.fr. Tel: +33 442 25 34 78

Content: Table S1: List of protein sequences used in this study; Table S2: List of mutations, primer pairs, starting plasmids, resulting plasmids and resulting biosensors; Table S3: ΔR/R calculated for biosensor Cit Δ1Δ2Δ3Δ4; Figure S1: Speciation diagram for a solution containing uranyl, IDA and biosensor 1 + fitting curves for uranyl-biosensor 1 and uranyl-biosensor Cit; Figure S2: Fluorescence spectra of biosensor Δ1S2I with calcium or uranyl.

Protein Name	Protein sequence
Biosensor 1	SMVSKGEELFTGVVPILVELDGDVNGHRFSVSGEGEGDATYGKLTLKFICTTGKLPVPWPTLVTTLTWGVQCFSRYP<br>DHMKQHDFFKSAMPEGYVQERTIFFKDDGNYKTRAEVKFEGDTLVNRIELKGIDFKEDGNILGHKLEYNYISHNVYIT<br>ADKQKNGIKAHFKIRHNIEDGSVQLADHYQQNTPIGDGPVLLPDNHYLSTQSALSKDPNEKRDHMVLLEFVTAAel<br>MADQLTDDQISEFKEAFSLFDKDGDGCITTKELGTVMRSLGQNPTEAELQDMINEVDADGNGTIDFPEFLNLMAR<br>KMKDTDSEEELKEAFRVFDKDQNGFISAAELRHVMTNLGEKLTDEEVDEMIKEADVDGDGQINYEEFVKVMMAK<br>**GGGGS**KRRWKKNFIAVSAANRFKKISSSGALvdMVSKGEELFTGVVPILVELDGDVNGHKFSVSGEGEGDATYGKL<br>TLKFICTTGKLPVPWPTLVTTFGYGVQCFARYPDHMKQHDFFKSAMPEGYVQERTIFFKDDGNYKTRAEVKFEGDT<br>LVNRIELKGIDFKEDGNILGHKLEYNYNSHNVYIMADKQKNGIKVNFKIRHNIEDGSVQLADHYQQNTPIGDGPVLL<br>PDNHYLSYQSALSKDPNEKRDHMVLLEFVTAAGITLGMDELYK
Biosensor Δ1	SMVSKGEELFTGVVPILVELDGDVNGHRFSVSGEGEGDATYGKLTLKFICTTGKLPVPWPTLVTTLTWGVQCFSRYP<br>DHMKQHDFFKSAMPEGYVQERTIFFKDDGNYKTRAEVKFEGDTLVNRIELKGIDFKEDGNILGHKLEYNYISHNVYIT<br>ADKQKNGIKAHFKIRHNIEDGSVQLADHYQQNTPIGDGPVLLPDNHYLSTQSALSKDPNEKRDHMVLLEFVTAAel<br>MADQLTDDQISEFKEAFSLFDGDGCITTKELGTVMRSLGQNPTEAELQDMINEVDADGNGTIDFPEFLNLMARKM<br>KDTDSEEELKEAFRVFDKDQNGFISAAELRHVMTNLGEKLTDEEVDEMIKEADVDGDGQINYEEFVKVMMAK**GG**<br>**GGS**KRRWKKNFIAVSAANRFKKISSSGALvdMVSKGEELFTGVVPILVELDGDVNGHKFSVSGEGEGDATYGKLTLK<br>FICTTGKLPVPWPTLVTTFGYGVQCFARYPDHMKQHDFFKSAMPEGYVQERTIFFKDDGNYKTRAEVKFEGDTLVN<br>RIELKGIDFKEDGNILGHKLEYNYNSHNVYIMADKQKNGIKVNFKIRHNIEDGSVQLADHYQQNTPIGDGPVLLPDN<br>HYLSYQSALSKDPNEKRDHMVLLEFVTAAGITLGMDELYK
Biosensor<br>Δ1Δ3	SMVSKGEELFTGVVPILVELDGDVNGHRFSVSGEGEGDATYGKLTLKFICTTGKLPVPWPTLVTTLTWGVQCFSRYP<br>DHMKQHDFFKSAMPEGYVQERTIFFKDDGNYKTRAEVKFEGDTLVNRIELKGIDFKEDGNILGHKLEYNYISHNVYIT<br>ADKQKNGIKAHFKIRHNIEDGSVQLADHYQQNTPIGDGPVLLPDNHYLSTQSALSKDPNEKRDHMVLLEFVTAAel<br>MADQLTDDQISEFKEAFSLFDGDGCITTKELGTVMRSLGQNPTEAELQDMINEVDADGNGTIDFPEFLNLMARKM<br>KDTDSEEELKEAFRVFDQNGFISAAELRHVMTNLGEKLTDEEVDEMIKEADVDGDGQINYEEFVKVMMAK**GGGG**<br>**S**KRRWKKNFIAVSAANRFKKISSSGALvdMVSKGEELFTGVVPILVELDGDVNGHKFSVSGEGEGDATYGKLTLKFIC<br>TTGKLPVPWPTLVTTFGYGVQCFARYPDHMKQHDFFKSAMPEGYVQERTIFFKDDGNYKTRAEVKFEGDTLVNRIE<br>LKGIDFKEDGNILGHKLEYNYNSHNVYIMADKQKNGIKVNFKIRHNIEDGSVQLADHYQQNTPIGDGPVLLPDNHYL<br>SYQSALSKDPNEKRDHMVLLEFVTAAGITLGMDELYK
Biosensor<br>Δ1Δ2Δ3	SMVSKGEELFTGVVPILVELDGDVNGHRFSVSGEGEGDATYGKLTLKFICTTGKLPVPWPTLVTTLTWGVQCFSRYP<br>DHMKQHDFFKSAMPEGYVQERTIFFKDDGNYKTRAEVKFEGDTLVNRIELKGIDFKEDGNILGHKLEYNYISHNVYIT<br>ADKQKNGIKAHFKIRHNIEDGSVQLADHYQQNTPIGDGPVLLPDNHYLSTQSALSKDPNEKRDHMVLLEFVTAAel<br>MADQLTDDQISEFKEAFSLFDGDGCITTKELGTVMRSLGQNPTEAELQDMINEVDGNGTIDFPEFLNLMARKMKD<br>TDSEEELKEAFRVFDQNGFISAAELRHVMTNLGEKLTDEEVDEMIKEADVDGDGQINYEEFVKVMMAK**GGGGS**KR

	RWKKNFIAVSAANRFKKISSSGALvdMVSKGEELFTGVVPILVELDGDVNGHKFSVSGEGEGDATYGKLTLKFICTTG<br>KLPVPWPTLVTTFGYGVQCFARYPDHMKQHDFFKSAMPEGYVQERTIFFKDDGNYKTRAEVKFEGDTLVNRIELKG<br>IDFKEDGNILGHKLEYNYNSHNVYIMADKQKNGIKVNFKIRHNIEDGSVQLADHYQQNTPIGDGPVLLPDNHYLSY<br>QSALSKDPNEKRDHMVLLEFVTAAGITLGMDELYK
Biosensor<br>Δ1Δ2Δ3Δ4	SMVSKGEELFTGVVPILVELDGDVNGHRFSVSGEGEGDATYGKLTLKFICTTGKLPVPWPTLVTTLTWGVQCFSRYP<br>DHMKQHDFFKSAMPEGYVQERTIFFKDDGNYKTRAEVKFEGDTLVNRIELKGIDFKEDGNILGHKLEYNYISHNVYIT<br>ADKQKNGIKAHFKIRHNIEDGSVQLADHYQQNTPIGDGPVLLPDNHYLSTQSALSKDPNEKRDHMVLLEFVTAAel<br>MADQLTDDQISEFKEAFSLFDGDGCITTKELGTVMRSLGQNPTEAELQDMINEVDGNGTIDFPEFLNLMARKMKD<br>TDSEEELKEAFRVFDQNGFISAAELRHVMTNLGEKLTDEEVDEMIKEADGDGQINYEEFVKVMMAK**GGGGS**KRR<br>WKKNFIAVSAANRFKKISSSGALvdMVSKGEELFTGVVPILVELDGDVNGHKFSVSGEGEGDATYGKLTLKFICTTGK<br>LPVPWPTLVTTFGYGVQCFARYPDHMKQHDFFKSAMPEGYVQERTIFFKDDGNYKTRAEVKFEGDTLVNRIELKGI<br>DFKEDGNILGHKLEYNYNSHNVYIMADKQKNGIKVNFKIRHNIEDGSVQLADHYQQNTPIGDGPVLLPDNHYLSYQ<br>SALSKDPNEKRDHMVLLEFVTAAGITLGMDELYK
Biosensor S2I	SMVSKGEELFTGVVPILVELDGDVNGHRFSVSGEGEGDATYGKLTLKFICTTGKLPVPWPTLVTTLTWGVQCFSRYP<br>DHMKQHDFFKSAMPEGYVQERTIFFKDDGNYKTRAEVKFEGDTLVNRIELKGIDFKEDGNILGHKLEYNYISHNVYIT<br>ADKQKNGIKAHFKIRHNIEDGSVQLADHYQQNTPIGDGPVLLPDNHYLSTQSALSKDPNEKRDHMVLLEFVTAAel<br>MADQLTDDQISEFKEAFSLFDKDGDGCITTKELGTVMRSLGQNPTEAELQDMINEV**A**A**A**GNGTIDFPEFLNLMAR<br>KMKDTDSEEELKEAFRVFDKDQNGFISAAELRHVMTNLGEKLTDEEVDEMIKEADVDGDGQINYEEFVKVMMAK<br>**GGGGS**KRRWKKNFIAVSAANRFKKISSSGALvdMVSKGEELFTGVVPILVELDGDVNGHKFSVSGEGEGDATYGKL<br>TLKFICTTGKLPVPWPTLVTTFGYGVQCFARYPDHMKQHDFFKSAMPEGYVQERTIFFKDDGNYKTRAEVKFEGDT<br>LVNRIELKGIDFKEDGNILGHKLEYNYNSHNVYIMADKQKNGIKVNFKIRHNIEDGSVQLADHYQQNTPIGDGPVLL<br>PDNHYLSYQSALSKDPNEKRDHMVLLEFVTAAGITLGMDELYK
Biosensor<br>Δ1S2I	SMVSKGEELFTGVVPILVELDGDVNGHRFSVSGEGEGDATYGKLTLKFICTTGKLPVPWPTLVTTLTWGVQCFSRYP<br>DHMKQHDFFKSAMPEGYVQERTIFFKDDGNYKTRAEVKFEGDTLVNRIELKGIDFKEDGNILGHKLEYNYISHNVYIT<br>ADKQKNGIKAHFKIRHNIEDGSVQLADHYQQNTPIGDGPVLLPDNHYLSTQSALSKDPNEKRDHMVLLEFVTAAel<br>MADQLTDDQISEFKEAFSLFDGDGCITTKELGTVMRSLGQNPTEAELQDMINEV**A**A**A**GNGTIDFPEFLNLMARKM<br>KDTDSEEELKEAFRVFDKDQNGFISAAELRHVMTNLGEKLTDEEVDEMIKEADVDGDGQINYEEFVKVMMAK**GG**<br>**GGS**KRRWKKNFIAVSAANRFKKISSSGALvdMVSKGEELFTGVVPILVELDGDVNGHKFSVSGEGEGDATYGKLTLK<br>FICTTGKLPVPWPTLVTTFGYGVQCFARYPDHMKQHDFFKSAMPEGYVQERTIFFKDDGNYKTRAEVKFEGDTLVN<br>RIELKGIDFKEDGNILGHKLEYNYNSHNVYIMADKQKNGIKVNFKIRHNIEDGSVQLADHYQQNTPIGDGPVLLPDN<br>HYLSYQSALSKDPNEKRDHMVLLEFVTAAGITLGMDELYK
Biosensor Cit	SMVSKGEELFTGVVPILVELDGDVNGHRFSVSGEGEGDATYGKLTLKFICTTGKLPVPWPTLVTTLTWGVQCFSRYP<br>DHMKQHDFFKSAMPEGYVQERTIFFKDDGNYKTRAEVKFEGDTLVNRIELKGIDFKEDGNILGHKLEYNYISHNVYIT<br>ADKQKNGIKAHFKIRHNIEDGSVQLADHYQQNTPIGDGPVLLPDNHYLSTQSALSKDPNEKRDHMVLLEFVTAAel<br>MADQLTDDQISEFKEAFSLFDKDGDGCITTKELGTVMRSLGQNPTEAELQDMINEVDADGNGTIDFPEFLNLMAR<br>KMKDTDSEEELKEAFRVFDKDQNGFISAAELRHVMTNLGEKLTDEEVDEMIKEADVDGDGQINYEEFVKVMMAK<br>**GGGGS**KRRWKKNFIAVSAANRFKKISSSGALvdMVSKGEELFTGVVPILVELDGDVNGHKFSVSGEGEGDATYGKL<br>TLKFICTTGKLPVPWPTLVTTFGYG**LM**CFARYPDHMKQHDFFKSAMPEGYVQERTIFFKDDGNYKTRAEVKFEGDT<br>LVNRIELKGIDFKEDGNILGHKLEYNYNSHNVYIMADKQKNGIKVNFKIRHNIEDGSVQLADHYQQNTPIGDGPVLL<br>PDNHYLSYQSALSKDPNEKRDHMVLLEFVTAAGITLGMDELYK
Biosensor Cit<br>Δ1Δ2Δ3Δ4	SMVSKGEELFTGVVPILVELDGDVNGHRFSVSGEGEGDATYGKLTLKFICTTGKLPVPWPTLVTTLTWGVQCFSRYP<br>DHMKQHDFFKSAMPEGYVQERTIFFKDDGNYKTRAEVKFEGDTLVNRIELKGIDFKEDGNILGHKLEYNYISHNVYIT<br>ADKQKNGIKAHFKIRHNIEDGSVQLADHYQQNTPIGDGPVLLPDNHYLSTQSALSKDPNEKRDHMVLLEFVTAAel<br>MADQLTDDQISEFKEAFSLFDGDGCITTKELGTVMRSLGQNPTEAELQDMINEVDGNGTIDFPEFLNLMARKMKD<br>TDSEEELKEAFRVFDQNGFISAAELRHVMTNLGEKLTDEEVDEMIKEADGDGQINYEEFVKVMMAK**GGGGS**KRR<br>WKKNFIAVSAANRFKKISSSGALvdMVSKGEELFTGVVPILVELDGDVNGHKFSVSGEGEGDATYGKLTLKFICTTGK<br>LPVPWPTLVTTFGYG**LM**CFARYPDHMKQHDFFKSAMPEGYVQERTIFFKDDGNYKTRAEVKFEGDTLVNRIELKGI<br>DFKEDGNILGHKLEYNYNSHNVYIMADKQKNGIKVNFKIRHNIEDGSVQLADHYQQNTPIGDGPVLLPDNHYLSYQ<br>SALSKDPNEKRDHMVLLEFVTAAGITLGMDELYK

Table S1: List of protein sequences used in this study. The parts written in blue correspond to the protein sequence of the donor eCFP, the parts written in black correspond to the protein sequence of the CaM, the parts written in pink correspond to the linker (L), the parts written in red correspond to the M13 peptide and the parts written in orange correspond to the protein sequence of the acceptor eYFP or Citrine. Mutations are written in bold and in green. The position where the deletions occurred has been underlined with a line. The metal-binding loops of CaM are highlighted in yellow.

Mutations made (deletion or substitution)	Primer pairs (sequence 5′→3′)<br>*F: forwards; R: reverse*	Starting plasmid	Resulting plasmid	Resulting biosensor
Deletion $\Delta_{2,3}$ in site 1	F: GGAAGCCTTCAGCTTATTCGACGGTGATGGTTGCATTACC<br>R: GGTAATGCAACCATCACCGTCGAATAAGCTGAAGGCTTCC	A3	A4	Biosensor Δ1
Deletion $\Delta_{2,3}$ in site 3	F: GCCTTCCGCGTTTTCGACCAGAACGGTTTCATCAGC<br>R: GCTGATGAAACCGTTCTGGTCGAAAACGCGGAAGGC	A4	A5	Biosensor Δ1Δ3
Deletion $\Delta_{2,3}$ in site 2	F: CATGATCAACGAAGTGGATGGTAACGGTACCATTGATTTCCCG<br>R: CGGGAAATCAATGGTACCGTTACCATCCACTTCGTTGATCATG	A5	A6	Biosensor Δ1Δ2Δ3
Deletion $\Delta_{2,3}$ in site 4	F: GAGATGATCAAAGAAGCGGATGGTGATGGTCAGATTAAC<br>R: GTTAATCTGACCATCACCATCCGCTTCTTTGATCATCTC	A6	A7	Biosensor Δ1Δ2Δ3Δ4
Inactivated site 2<br>D57A D59A[1]	F: GATCAACGAAGTGGCTGCTGCTGGTAACGGTACC<br>R: GGTACCGTTACCAGCAGCAGCCACTTCGTTGATC	A3	A8	Biosensor S2I
		A4	A9	Biosensor Δ1S2I
Mutation eYFP to Citrine<br>V69L Q70M[2]	F: TTCGGCTACGGCCTGATGTGCTTCGCCCGCTACCCC<br>R: GGGGTAGCGGGCGAAGCACATCAGGCCGTAGCCGAA	A3	A10	Biosensor Cit
		A7	A11	Biosensor Cit Δ1Δ2Δ3Δ4

Table S2: List of mutations, primer pairs, starting plasmids, resulting plasmids and resulting biosensors used in this study. [1]Numbering from the beginning of the CaM sequence; [2]Numbering from the beginning of the eYFP sequence

	Biosensor Cit Δ1Δ2Δ3Δ4
ΔR/R with 1 mM KCl	0 %
ΔR/R with 100 μM NaCl	0 %
ΔR/R with 100 μM $MgCl_2$	4 %

Table S3: ΔR/R calculated with the Equation 4 for biosensor Cit Δ1Δ2Δ3Δ4 in presence of 1 mM KCl or 100 μM NaCl or 100 μM $MgCl_2$. These values resulted from the average of at least two independent experiments.

Figure S1

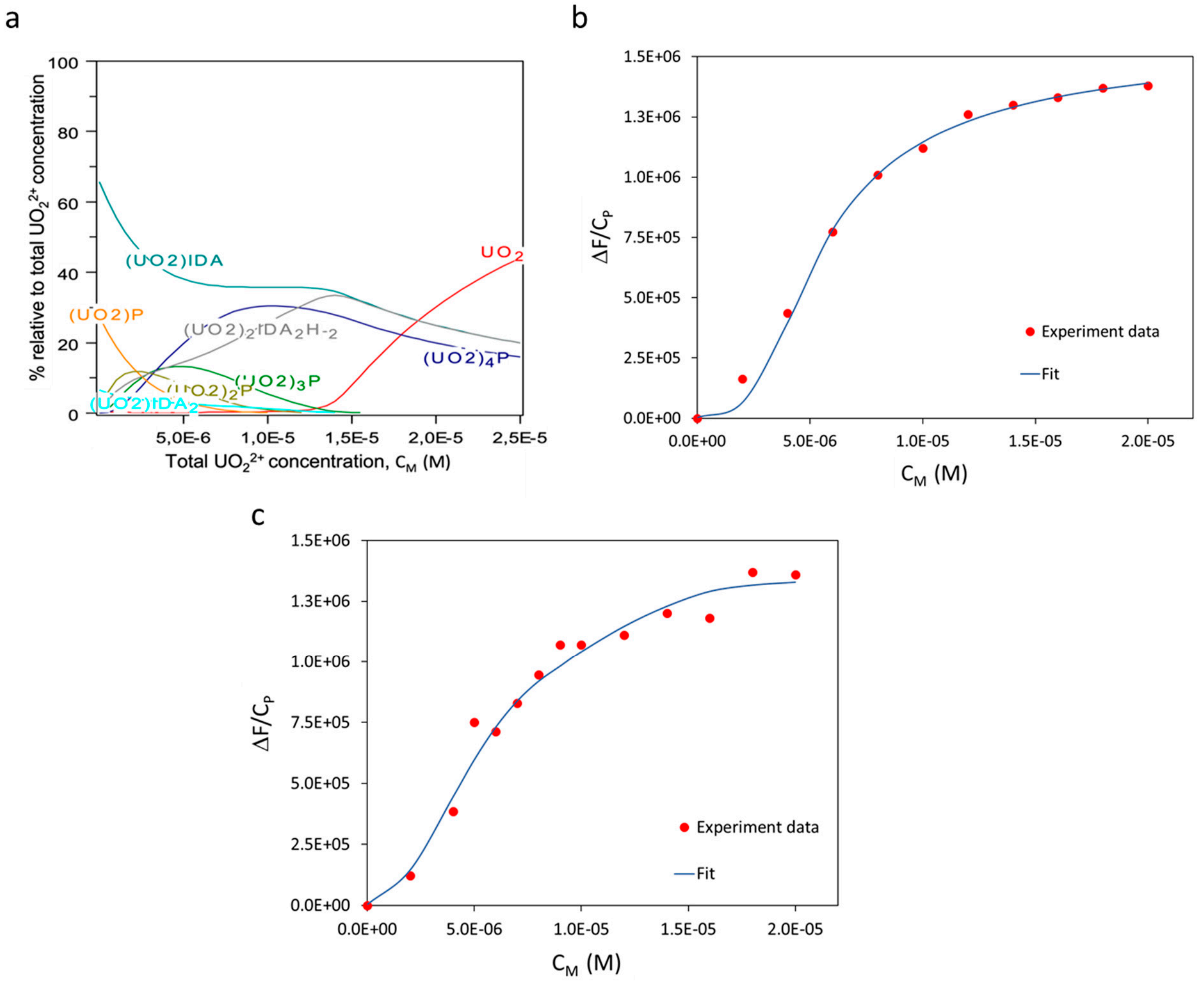

Figure S1: (a) Speciation diagram for a solution containing IDA at total concentration C_{IDA} = 10 µM, and biosensor 1 at total concentration C_P = 1 µM, pH = 7, the total uranyl concentration ranges between C_M = 0 and C_M = 20 µM. In the metal complex names, the protein is indicated as P. The stability constants used for the uranyl-IDA complexes are taken from Jiang et al. [35]. (b) Experimental data and corresponding fit for the uranyl-biosensor 1 titration (for the x axis legend, M = UO_2^{2+}). The fitting curve was obtained by using the equation 3: $\Delta F/C_P = \Delta\phi\, K_{cond}\, [M]^4 / (1 + K_{cond}\, [M]^4)$ and the algorithm described in Materials and Methods section of the manuscript. Input data: total concentrations of metal ion, protein and IDA, and ΔF, the change in fluorescence ratio (F_{525nm}/F_{476nm}) during titration. (c) Experimental data and corresponding fit for the uranyl-biosensor Cit titration. The fitting curve was obtained as explained above for (b).

Figure S2

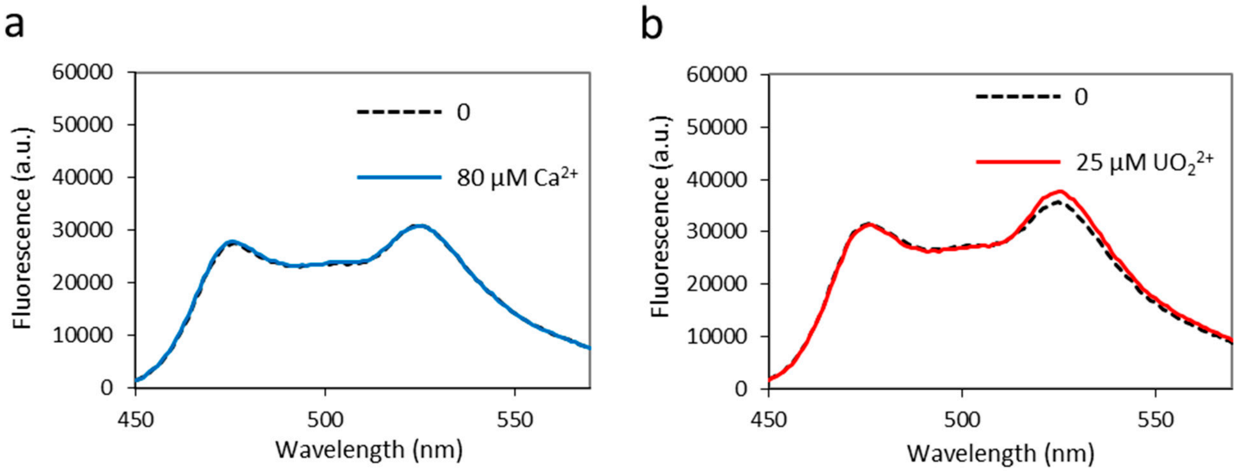

Figure S2: Fluorescence spectra of biosensor Δ1S2I with calcium (a) or uranyl (b) obtained with an excitation wavelength of 440 nm. Fluorescence measured in arbitrary units (a.u.) with no metal (dashed lines) and 80 µM of calcium or 25 µM of uranyl (solid lines).
